# ASPIRIN BENEFIT IS NOT AFFECTED BY BODY WEIGHT IN HEALTHY OLDER PERSONS

**DOI:** 10.1093/geroni/igz038.480

**Published:** 2019-11-08

**Authors:** Robyn L Woods, Galina Polekhina, Mark Nelson, Rory Wolfe, Michael E Ernst, Anne M Murray, John McNeil

**Affiliations:** 1 Monash University, Melbourne, Victoria, Australia; 2 University of Tasmania, Hobart, Tasmania, Australia; 3 Monash University, Melbourne, Australia; 4 University of Iowa College of Pharmacy, Iowa City, Iowa, United States; 5 Berman Center for Outcomes and Clinical Research, Minneapolis, Minnesota, United States; 6 Department of Epidemiology & Preventive Medicine, Monash University, Melbourne VIC, Australia, Melbourne VIC, Australia, Australia

## Abstract

Recent meta-analysis (Rothwell et al, Lancet 2018;392:387-99) of aspirin trials in predominantly middle-aged people suggested that aspirin’s effects on clinical outcomes vary according to dose and body weight, concluding that under dosing in heavier people may be responsible for reduced efficacy of aspirin. Data from ASPREE (ASPirin in Reducing Events in the Elderly; randomized primary prevention trial of low dose aspirin versus placebo in 19,114 healthy older participants, mainly aged 70+ years) were analyzed for interaction of body habitus on the main outcomes after 4.7 years of study treatment. Increases in body weight, BMI or waist circumference (WC) did not influence cardiovascular endpoints or incident cancers in the aspirin group compared with placebo. In ASPREE men, an increase of 10 kg body weight elevated the risk of major hemorrhage with aspirin (HR 1.20; 95% confidence interval 1.01-1.43; P=0.04) and 10 cm increase in WC elevated all-cause mortality by 23% with aspirin (HR 1.23; 95% confidence interval 105-1.44; P=0.01), driven by cancer-related deaths (HR 1.39; 95% confidence interval 1.11-1.73; P=0.004). These effects of increased abdominal girth were not seen in women. Evidence from ASPREE does not support increasing the dose of aspirin in larger older people to improve the drug’s efficacy. Our results point to a link between central adiposity, hemorrhage risk and cancer-related death in older men taking aspirin.

